# Sexually Transmitted Infections in Women Participating in a Biomedical Intervention Trial in Durban: Prevalence, Coinfections, and Risk Factors

**DOI:** 10.1155/2013/358402

**Published:** 2013-11-03

**Authors:** Nathlee Samantha Abbai, Handan Wand, Gita Ramjee

**Affiliations:** ^1^HIV Prevention Research Unit, Medical Research Council, Durban 4000, South Africa; ^2^The Kirby Institute, University of New South Wales, Sydney, NSW, Australia; ^3^Department of Epidemiology and Population Health, London School of Hygiene & Tropical Medicine, London, UK

## Abstract

*Background*. Sexually transmitted infections (STIs) continue to be a significant public health problem especially among women of reproductive age in Africa. *Methods*. A total of 2236 women that had enrolled in the MDP301 vaginal microbicide trial were tested for the presence of *Chlamydia trachomatis* (CT), *Neisseria gonorrhea* (NG), *Treponema pallidum,* and *Trichomonas vaginalis* (TV). *Results*. CT was identified as the most prevalent STI (11%) followed by TV (10%), NG, and Syphilis (3%). The highest prevalence of coinfection was reported between *T. pallidum* and TV (19.67%, *P* = 0.004), followed by CT and TV (13.52%, *P* ≤ 0.001). Risk factors that were significantly associated with STI acquisition were women of 23 years of age or younger (HR: 1.50, 95% CI 1.17, 1.93), baseline STI with CT (HR: 1.77, 95% CI 1.32, 2.35), TV (HR: 1.58, 95% CI, 1.20, 2.10), and *T. pallidum* (HR: 5.13, 95% CI 3.65, 7.22), and a low education level (HR: 1.30, 95% CI 1.02, 1.66). *Conclusion*. Young women with lower education and a history of STIs are at high risk of multiple STIs. Prevention programs should consider target approach to STI prevention among young women. This trial is registered with ISRCTN64716212.

## 1. Introduction

According to the World Health Organization (WHO) 2008 estimates, the total global number of new cases of four of the curable sexually transmitted infections (STIs), *Chlamydia trachomatis* (CT)*, Neisseria gonorrhoeae* (NG), *Treponema pallidum* (Syphilis), and *Trichomonas vaginalis* (TV), was nearly 500 million cases in adults aged 15 to 49 years (WHO) [1]. In Sub-Saharan Africa alone, 69 million cases of these STIs are reported annually [2]. STIs continue to be a significant public health problem with an increased burden on women of reproductive age [3, 4]. They have been associated with a wide spectrum of complications such as urethritis and epididymitis in men and cervicitis in women [5, 6]; thus the more ascending cervical infections may cause pelvic inflammatory disease and untimely tubal pathology, which increases the risk of ectopic pregnancy, tubal infertility, and chronic abdominal pain [7, 8].

According to the WHO and UNAIDS, STI control programmes have three objectives: to interrupt the transmission of STIs; to prevent the development of diseases, complications, and sequelae; and to reduce the transmission of HIV infection [9]. Treatment for STIs needs to be effective and administered as promptly as possible. The syndromic management approach allows for immediate treatment based on the presentation of signs and symptoms. 

In South Africa, STIs including human immunodeficiency virus (HIV) contribute to the disease burden [10]. Additionally, the prevalence of STIs is higher in South Africa when compared to other African countries [11] accounting for 11 million cases being treated annually [12]. According to recent South African sentinel surveillance data, the prevalence of some STIs such as CT and NG has not declined since 2003 [13]. Although syndromic case management remains the foundation for treatment of STIs in many countries around the world, including South Africa [14], there are, however, various limitations of syndromic management including failing to treat asymptomatic infections, overtreatment, and poor sensitivity and specificity of algorithms in correctly diagnosing the infections, particularly for women [8]. Therefore, the monitoring of STI prevalence adds value to the evaluation of treatment interventions, and it provides an indirect measure of change in sexual behavior [10]. 

This study reports of the prevalence STIs among a cohort of young women and the associated risk factors which increase the women's risk of acquiring new infections (incidence/point prevalence).

## 2. Methods

### 2.1. Study Sites

Data was analyzed from 2236 sexually active women who participated in the Microbicides Development Programme (MDP), MDP 301 trial conducted between 2005 and 2009, described in detail elsewhere [11, 12]. The trial was an international, multicenter, randomized, double-blind, placebo controlled trial to assess the safety and efficacy of 0.5% and 2% PRO 2000/5 gels for the prevention of vaginally acquired HIV infection. The products were not found to be effective in preventing STIs. The study enrolled women from six research sites in Africa (three South African sites (Durban, Northern KwaZulu-Natal, and Johannesburg), Tanzania, Uganda, and Zambia). However, this analysis is based on participants recruited and enrolled at the Durban sites (Tongaat, Verulam, and Isipingo).

### 2.2. Study Population

For this study, women were recruited from health centers, family planning clinics, and urban settlements. Women were also recruited through community meetings and door-to-door visits. 

Women were eligible for the study participation if they met the following criteria: 18 years of age and above at enrolment; willing and able to give informed consent; HIV negative; nonpregnant with no intention to become so; sexually active and willing to use the study gel as instructed; and anticipating residence in and around the study area. Women were ineligible if they had had any severe clinical, laboratory, or gynecological abnormalities and were unlikely to comply with the protocol. Women were also deemed ineligible if they had sex >14 times per week based on prior data that suggested more frequent sex, and associated use of gels actually increases risk of HIV/STIs [15]. Participants were enrolled in the trial if they satisfied all the eligibility criteria and provided written informed consent. Women participating in the trial were provided with HIV testing and counseling, free condoms, risk reduction education, and diagnosis and treatment of STIs. Counselors emphasized that condoms are the only known method to prevent acquisition of HIV and STIs and that condoms should be used for every sex act. 

### 2.3. Study Procedures

During screening, information on demographic characteristics and other information that is required to determine eligibility were collected from women. For those women meeting the eligibility criteria and being enrolled in the study, information on baseline sexual practice and behaviour as well as medical and menstrual history was obtained. Additionally, behavioural questionnaires focusing on frequency of condom use and frequency of sex acts were administered by trained interviewers via in-depth interviews and focus group discussion. 

STI testing was conducted on swabs and blood samples. Samples were collected by study nurses at enrollment and week 24 of the follow-up visit. CT and NG infections were assessed using amplicor swabs collected during the genital examination by using nucleic acid amplification assays (COBAS Amplicor, Roche Molecular Diagnostics, Pleasanton, CA, USA). Baseline and confirmatory *T. pallidum* testing was conducted on sera using the RPR (BD Macrovue) and TPHA (Fujibrio) methods, respectively. Testing for TV was conducted using sterile swabs with the In-Pouch-TV test kit. Participants were treated for curable STIs prior to enrollment and assessed and treated for incident infections during study follow-up.

The protocol was approved by local and international ethics and regulatory bodies. For the Durban sites ethical oversight for the study was provided by the Biomedical Research Ethics Committee based at the University of KwaZulu-Natal. The study was also approved by the Medicines Control Council in South Africa. 

### 2.4. Statistical Analyses

Frequency distribution and percentages were used to describe the demographic and socioeconomic characteristics of the study population. Variables included in this analysis were age, education level, employment status (employed/regular income versus not), and being diagnosed with any bacterial STI at baseline. Cox proportional hazard regression models were used to assess the predictors of being diagnosed with an STI. The presence of STI coinfections was compared by the chi-square test to determine if there was any relation between two STIs. Time-dependent measures analyzed were biological factors such as history of having any bacterial STI (CT, NG*, T. pallidum,* or TV). Multivariate models considered all variables statistically significant (*P* < 0.10) in the initial analyses and used forward stepwise method. Statistical analysis was performed using STATA release 12.0 (College Station, Texas, TX, USA). 

## 3. Results 

Of the 2236 women included in this study, 492 (22%) were diagnosed with an STI at baseline. The most prevalent STI in this cohort was CT which was detected in a total of 244/2236 (11%) followed by TV in 200/2236 (10%), NG in 71/2236 (3%), and *T. pallidum* in 56/2236 (3%). The prevalence of STI coinfections is represented in Table 1. The highest prevalence of coinfection was observed between TV and *T. pallidum* (19.67%, *P* = 0.004) followed by CT and TV (13.52%, *P* = 0.001) and CT and NG (12.70%, *P* = 0.001). Only women who tested positive for TV were coinfected with *T. pallidum*. There was no coinfection reported between *T. pallidum* with CT or NG.

Table 2 presents the baseline characteristics of the women with or without any bacterial STI. At baseline, younger women (<23 years) had a higher prevalence of at least one STI as compared to older women (>38) (37% and 19%, *P* ≤ 0.001) showing that there is a decreasing STI prevalence with increasing age thus representing an inversely proportional trend between age and STI prevalence. However, at baseline testing positive for STIs was not associated with employment status (80%, *P* = 0.219), education status (80%, *P* = 0.50), condom use at last sex act (60%, *P* = 0.221), having more than 3 sexual acts in the past two weeks (43%, *P* = 0.219), and hormonal contraception (62%, *P* = 0.7).

### 3.1. Risk Factors for STI Acquisition

Table 3 presents the results of unadjusted and adjusted analysis (i.e., Cox regression) with the incidence rates represented as hazard ratios (HR). In this study, the overall crude STI incidence rate was reported to be 17 per 100 person-years (95% confidence interval (CI) 15.3, 18.8). 

The unadjusted analysis showed that, being 23 years of age or younger (HR 1.36, 95% CI 1.09, 1.71), baseline STI with CT (HR: 1.77, 95% CI 1.35, 2.31), TV (HR: 1.89, 95% CI 0.43, 2.50), and *T. pallidum* (HR: 4.67, 95% CI 3.37, 6.46) and a low education level (HR: 1.27, 95% CI 1.00, 1.60) were all significantly associated with increased risk of STI acquisition.

Similarly, in the adjusted analysis, being 23 years of age or younger (HR: 1.50, 95% CI 1.17, 1.93) put women at higher STI acquisition when compared to older age groups, 23 to 27 (HR: 1.07, 95% CI 0.80, 1.44), 28 to 32 (HR: 0.83, 95% CI 0.63, 1.10), and 38 years and older. Additionally, a higher STI incidence rate was reported in women 23 years and younger 21 per 100 person years, 95% CI 17.8, 24) when compared to the oldest age (38 years and older) group in which the STI incidence was reported to be 17 per 100 person years (95% CI 14.4, 21.7).

Other risk factors that were significantly associated with STI acquisition were baseline STI with CT (HR: 1.77, 95% CI 1.32, 2.35), TV (HR: 1.58, 95% CI 1.20, 2.10), and *T. pallidum* (HR: 5.13, 95% CI 3.65. 7.22) and a low education level (HR: 1.30, 95% CI 1.02, 1.66). No significant associations were found with baseline diagnosis of NG and risk for STI acquisition in both the unadjusted (HR: 1.49, 95% CI 0.92, 2.43) and adjusted analyses (HR: 1.19, 95% CI 0.72, 1.98), respectively. 

## 4. Discussion

The province KwaZulu-Natal (KZN) is one of the most densely populated regions of South Africa accounting for 21% of the total population; of these 59% of the total population is reported to be sexually active. The prevalence of each of the STIs reported at enrollment was fairly high with CT being the most prevalent STI, followed by TV, NG, and Syphilis, respectively. The data presented in this study is comparable with those presented by Johnson et al. [10] who showed that CT and TV infections are more prevalent in the general population when compared to prevalence of NG and *T. pallidum*. The lower prevalence of NG and *T. pallidum* reported in this study could be attributed to the fact that these women were from local communities and not high risk women such as sex workers. Since the introduction of syndromic approach of STI management there have been reports of declining STI rates in South Africa in some regions [13]. However, in KZN, incidence of STIs remains high with 26.7 cases per 100 person years [10]. Studies among pregnant women showed coinfection between the 40% of pregnant women that have TV infection; almost 2–17% are coinfected with *T. pallidum* [13, 16]. This was not the case among nonpregnant women in this study. In the trials conducted, pregnancy was an exclusionary criterion. However, the observed high percentage of coinfection between CT and TV is of interest. Additionally, the observed high percentage of coinfection between CT and TV proves to be interesting. Swygard et al. [17] reported coinfection rates between CT and TV to be 15–28% in the USA. In South Africa, coinfection rates for CT and TV have only been reported in men with urethritis [18] and pregnant women [19], thereby limiting the comparison of our study data with other reports from South Africa. To the best of our knowledge, our data is novel in nonpregnant women and suggests that younger women whether pregnant or not have a higher prevalence of STIs. 

More importantly the study also identified risk factors for incident infections. Younger women were more at risk of acquiring new STIs. The reasons for younger women being more susceptible to infection can be associated with biological and behavioral factors. In younger women, the epithelial cells of the immature cervix are more vulnerable to infection [20]. STIs such as CT have an affinity for infecting columnar epithelial cells, and younger women have greater areas of columnar epithelium on the ectocervix making them more susceptible to infection [21]. Furthermore age of sexual debut is also reported to be associated with STIs [22].

Being previously diagnosed with an STI was also an associated risk factor for incident infection as evidenced in this study. Young women are especially at risk for reinfection [23], since they may lack the confidence and power to negotiate safer sex practices. Women that repeatedly acquire STIs place themselves at risk for infection sequelae such as pelvic inflammatory diseases and ectopic pregnancy. Furthermore, when compared to older women, younger women are more prone to risky sexual behavior such as having multiple partners and poor condom negotiation skills [3, 4]. Cudmore and Garber [24] also reported that the most common risk factors for acquiring an STI included low level of education and having a history of an STI.

It was evident in this study that poorly educated women are at risk for infection. A similar observation was made by Solomon et al. [25] who investigated the relationship between formal education and risk for acquiring STIs in a population of high risk women. This study provided evidence that low level education is indeed a risk factor for infection. These findings were confirmed by Faber et al. [20] and Kenyon and Badri [22].

Although women were provided with condoms and safe sex education our data suggests that social desirability bias may be the case where women reported higher condom use in such research settings. This highlights the lack of condom negotiation power by women. Studies have reported that consistent condom use has been shown to reduce, but not eliminate, the acquisition of NG, CT, genital herpes, and *T. pallidum* in men and women, as well as human papilloma virus (HPV) and TV infections in women [26]. 

## 5. Limitations

The data presented in this study is limited to the population in which the research was conducted and may not be representative of women in the general population. Although the data on behavioral and demographic characteristics were collected by trained interviews to minimize bias and social desirability, misreporting could have skewed the data. In addition, documented STI rates in this study group may have been affected (i.e., decreased) study's educational and preventive interventions at study enrollment (e.g., lower risk for STIs than in similarly aged nontrial populations).

## 6. Conclusion

Young women in South Africa remain at a very high risk of acquiring multiple STIs which in turn puts them at risk of other diseases including the risk of HIV acquisition. We identified specific risk factors in our community of women, and they should be considered for targeted interventions among young women. More importantly it is critical to have point-of-care testing for ease of accurate treatment to minimize microbial resistance in settings of high STI prevalence and syndromic approach to treatment. The use of rapid point-of-care (POC) diagnostic tests could lead to more accurate STI testing and appropriate treatment [25]. It is therefore critical that community based clinics are empowered to conduct point-of-care testing in high STI endemic areas.

Routine testing of STIs is encouraged for the identification and management of coinfections especially in women, since the combined pathogenesis of CT, TV, NG, and *T. pallidum* can lead to severe reproductive complications. The identification of risk factors for infection will act as a cornerstone for future prevention efforts.

Prevention efforts should be directed toward a more effective, economically feasible approach which is scalable to a population level.

## Figures and Tables

**Table 1:** Coinfection among sexually transmitted infections reported in this study.

	STI negative *N* (%)	STI positive *N* (%)	*P* value
	CT negative	CT positive	

All	**1,992**	**244**	—
NG positive	40 (2.01)	31 (12.70)	<0.001
TV positive	167 (8.38)	33 (13.52)	<0.001
Syphilis positive	56 (2.8)	—	—

	*T. pallidum *negative	*T. pallidum *positive	

All	**2,180**	**56**	
NG positive	71 (3.26)	—	—
TV positive	189 (8.67)	11 (19.67)	0.004

	NG negative	NG positive	

All	**2,165**	**71**	
TV positive	191 (8.8)	9 (12.7)	0.263

CT: *Chlamydia trachomatis*, TV: *Trichomonas vaginalis*, NG: *Neisseria gonorrhoeae*.

**Table 2:** The association between demographic factors and prevalence of sexually transmitted infections.

Characteristics	Overall	STI−	STI+^*^	*P* value
	*N* = 2236 (%)	*N* = 1744 (%)	*N* = 492 (%)	
Age				<0.001
<23 years	660 (30)	477 (28)	183 (37)	
23–27 years	484 (22)	376 (22)	108 (22)	
28–32 years	564 (25)	454 (26)	110 (22)	
38+ years	528 (24)	437 (25)	91 (19)	
Education				0.584
At least high school				
No	1550 (69)	1204 (69)	346 (70)	
Yes	686 (30)	540 (31)	146 (30)	
Regular income/employment				0.212
No	1842 (82)	1446 (83)	396 (80)	
Yes	394 (18)	298 (17)	96 (20)	
Sexual behaviour				0.219
<3 sexual acts reported in the last 2 weeks	1282 (57)	988 (56)	294 (60)	
3+ sexual acts reported in the last 2 weeks	954 (43)	756 (43)	198 (40)	
Condom use at last sex act				0.221
No	896 (40)	687 (39)	209 (42)	
Yes	1339 (60)	1056 (60)	283 (57)	
Hormonal contraception^a^				0.75
No	845 (37)	662 (37)	183 (37)	
Yes	1391 (62)	1082 (62)	309 (62)	

^*^At least one positive test for *Chlamydia trachomatis*, *Neisseria gonorrhoeae*, *Trichomonas vaginalis,* and *T. pallidum* at enrolment.

^a^includes injectable and oral contraception.

**Table 3:** Risk factors for STI acquisition during study follow-up.

	STI incidence			
	Unadjusted HR (95% CI)	*P* value	Adjusted^a^ HR (95% CI)	*P* value
Age				
<23 years	1.36 (1.09, 1.71)	0.007	1.50 (1.17, 1.93)	0.001
23–27 years	0.95 (0.72, 1.26)	0.720	1.07 (0.80, 1.44)	0.628
28–32 years	0.84 (0.63, 1.10)	0.191	0.83 (0.63, 1.10)	0.197
38+ years	1		1	
Previous STI diagnosis				
*Chlamydia *	1.77 (1.35, 2.31)	0.000	1.77 (1.32, 2.35)	0.000
Gonorrhea	1.49 (0.92, 2.43)	0.104	1.19 (0.72, 1.98)	0.500
*Trichomonas *	1.89 (1.43, 2.50)	0.000	1.58 (1.20, 2.10)	0.001
*T. pallidum *	4.67 (3.37, 6.46)	0.000	5.13 (3.65. 7.22)	0.000
Education				
Low education	1.27 (1.00, 1.60)	0.044	1.30 (1.02, 1.66)	0.034

CI: confidence interval; HR: hazard ratio.
